# The Role of the Body Clock in Asthma and COPD: Implication for Treatment

**DOI:** 10.1007/s41030-018-0058-6

**Published:** 2018-06-01

**Authors:** Karolina Krakowiak, Hannah J. Durrington

**Affiliations:** 1 Division of Infection, Immunity and Respiratory Medicine, School of Biological Sciences, Faculty of Biology, Medicine and Health, University of Manchester, Manchester, M13 9PT UK; 2 Wythenshawe Hospital, Manchester University NHS Foundation Trust, Southmoor Road, Wythenshawe, Manchester, M23 9LT UK

**Keywords:** Asthma, BMAL-1, Chronotherapy, Circadian, Clock, COPD, REV-ERB alpha

## Abstract

Asthma exhibits a marked time of day variation in symptoms, airway physiology, and airway inflammation. This is also seen in chronic obstructive pulmonary disease (COPD), but to a lesser extent. Our understanding of how physiological daily rhythms are regulated by the circadian clock is increasing, and there is growing evidence that the molecular clock is important in the pathogenesis of these two airway diseases. If time of day is important, then it follows that treatment of asthma and COPD should also be tailored to the most efficacious time of the day, a concept known as ‘chronotherapy’. There have been a number of studies to determine the optimal time of day at which to take medications for asthma and COPD. Some of these agents are already used ‘chronotherapeutically’ in practice (often at night-time). However, several studies investigating systemic and inhaled corticosteroids have consistently shown that the best time of day to take these medications for treating asthma is in the afternoon or early evening and not in the morning, when these medications are often prescribed. Future, large, randomized, placebo-controlled studies of systemic and inhaled corticosteroids in asthma and COPD are needed to inform clinical practice.

**Digital Features:**

This article is published with a graphical abstract to facilitate understanding of the article. To view digital features for this article go to the Supplementary Information of the article.

**Electronic Supplementary Material:**

Supplementary material is available for this article at 10.1007/s41030-018-0058-6 and is accessible for authorized users.

## Introduction

Over the past 15 years, our understanding of the body clock and biological rhythms has increased immeasurably. In 2017, the Nobel Prize in Physiology or Medicine was awarded jointly to Jeffrey C. Hall, Michael Rosbash, and Michael W. Young “for their discoveries of molecular mechanisms controlling the circadian rhythm” [1–3]. The circadian clock is crucial in regulating daily physiological processes and it is now realized that the time at which our immune system is triggered (by infection [4], vaccination [5], surgery [6]), appears to be critical to the way we respond to these insults. Several inflammatory diseases, such as asthma, display a marked time of day pattern in symptoms. Synchronizing drug treatment concentrations to rhythms in disease activity, to increase efficacy as well as to reduce adverse effects, is called chronotherapy. In this review article, we will discuss recent advances in our understanding of circadian biology, and how this relates to the treatment and management of asthma and chronic obstructive pulmonary disease. This article is based on previously conducted studies and does not contain any studies with human participants or animals performed by any of the authors.

## What is the Circadian (Body) Clock?

Our body clock allows us to generate circadian rhythms. Circadian rhythms (circa = about, dies = day) are patterns of behavior and physiology that follow a 24-h cycle. Circadian rhythms are autonomous, self-sustained oscillations in biologic processes entrained to environmental cues, the most important being light [7]. The ability to generate circadian rhythms enables us to anticipate environmental changes and optimize our survival.

### How are Circadian Rhythms Regulated?

**Fig. 1:**
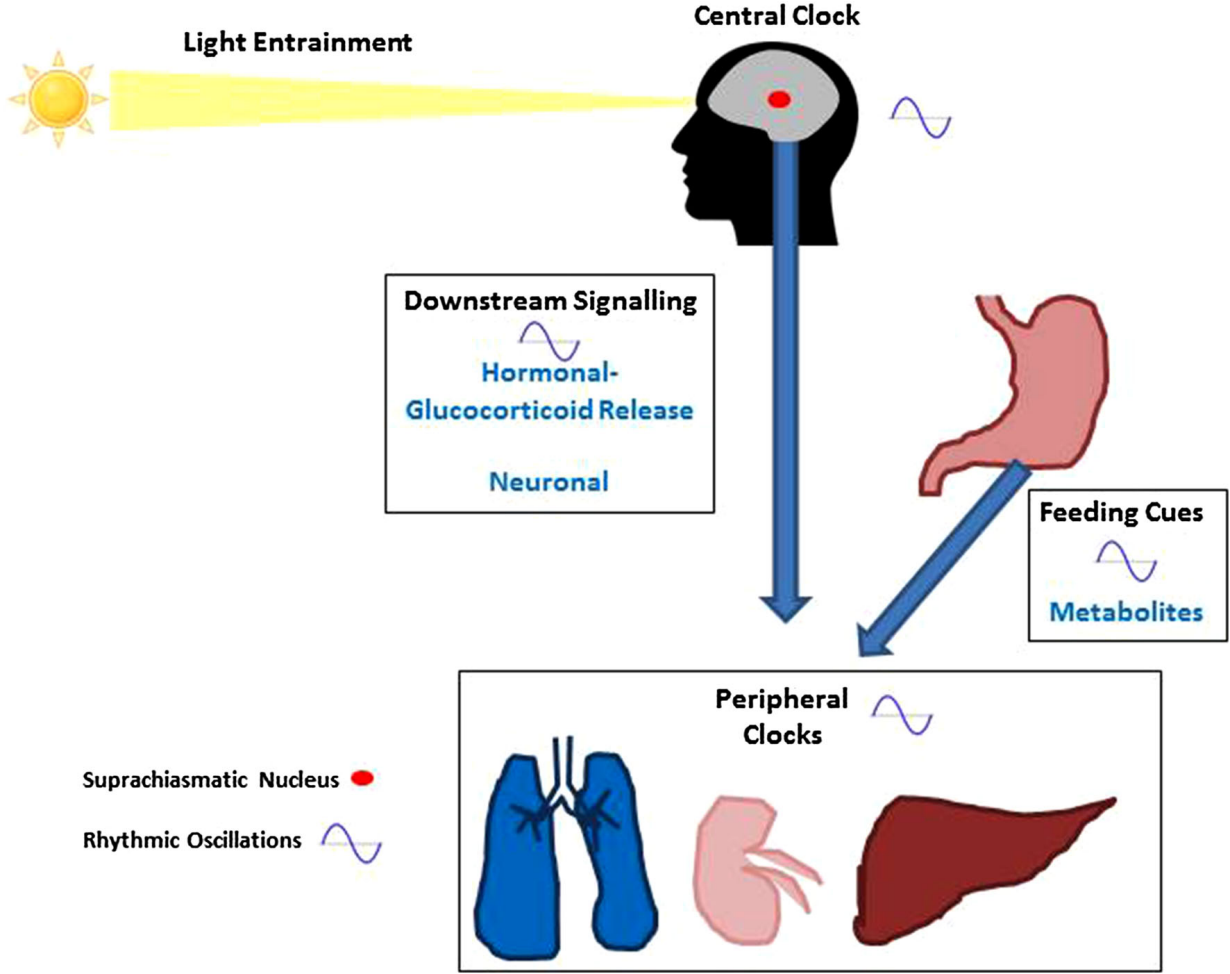
The central and peripheral clocks. The ‘central’ clock or pacemaker in the suprachiasmatic nucleus (SCN) of the brain integrates light and dark information and relays the information downstream to ‘peripheral’ clocks found in virtually every cell in the body, by a network involving neural pathways, hormone release (glucocorticoids), and metabolic cues from rhythmic feeding behavior. Light is the key entrainment factor for the SCN and feeding-regulated metabolic cues are pivotal for the regulation of many peripheral clocks

Circadian rhythmicity at a cellular level consists of the molecular clock, made up of a group of clock proteins that oscillate in a transcriptional–translational feedback loop. Each of these ‘peripheral’ clocks can track light and dark through messages received from the ‘central clock’ or pacemaker in the suprachiasmatic nucleus of the brain. The central pacemaker integrates light and dark information and relays the information downstream by a network involving neural pathways, hormone release (glucocorticoids), and metabolic cues from rhythmic feeding behavior [8, 9]. Light is the key entrainment factor for the SCN and feeding-regulated metabolic cues are pivotal for the regulation of many peripheral clocks [8, 9] Fig. 1.

Both central and peripheral clocks use the same molecular machinery to “time” the day. Interlocking repressing and activating transcriptional and translational feedback loops culminate in the approximately 24-h rhythmic expression and activity of a set of core clock genes in each organ.

CLOCK and BMAL1 increase transcription of period (PER1/2) and cryptochrome (CRY1/2) genes. As protein levels increase, PER and CRY associate and translocate into the nucleus, repressing CLOCK/BMAL1, thereby inhibiting their own transcription. Enzymatic degradation of PERIOD and CRYPTOCHROME proteins provides a delay mechanism prior to the onset of the next transcriptional cycle. The expression of positive factors, CLOCK and BMAL1, and negative factors, PER and CRY, are in antiphase to one another, providing circadian timing at the molecular level.

**Fig. 2:**
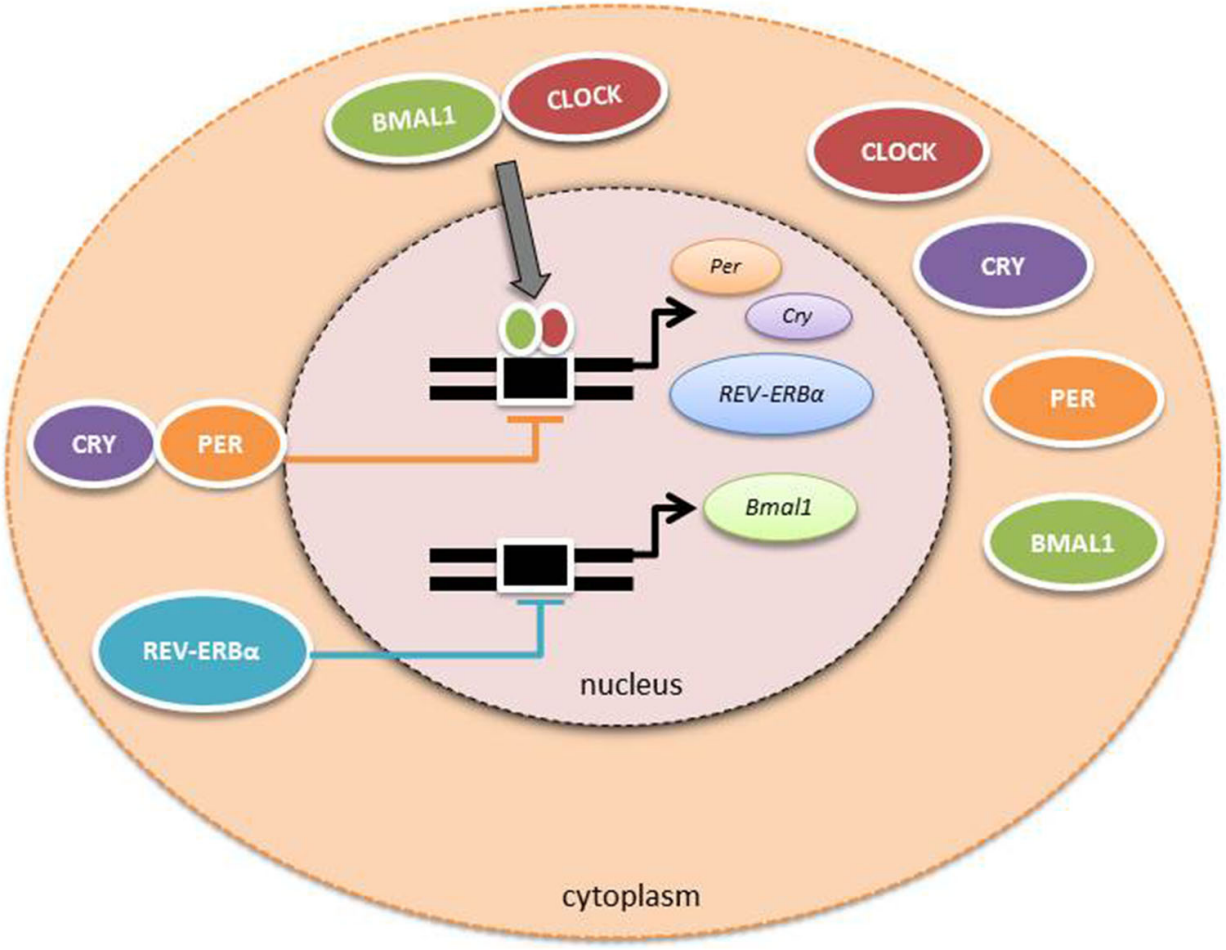
The molecular circadian clock. Both central and peripheral clocks use the same molecular machinery to “time” the day. Interlocking repressing and activating transcriptional and translational feedback loops culminate in the approximately 24-h rhythmic expression and activity of a set of core clock genes in each organ. CLOCK and BMAL1 increase transcription of period (PER1/2) and cryptochrome (CRY1/2) genes. As protein levels increase, PER and CRY associate and translocate into the nucleus, repressing CLOCK/BMAL1, thereby inhibiting their own transcription. Enzymatic degradation of PERIOD and CRYPTOCHROME proteins provides a delay mechanism prior to the onset of the next transcriptional cycle. The expression of positive factors, CLOCK and BMAL1, and negative factors, PER and CRY, are in antiphase to one another, providing circadian timing at the molecular level. Outputs from the molecular clock are generated through transcription or repression of target genes. BMAL1 is regulated by rhythmic interaction with REV-ERBα

Outputs from the molecular clock are generated through transcription or repression of target genes. BMAL1 is regulated by rhythmic interaction with REV-ERBα. REV-ERBα, a nuclear hormone receptor and core clock gene, is a critical regulator of inflammation and metabolism. REV-ERBα function can be regulated by small-molecule ligands and thus represents an exciting option for manipulation of the clock in disease states [10, 11] Fig. 2.

## What is Known About the Peripheral Lung Clock?

Work in our laboratory has shown that the peripheral lung clock is present in the Club cell in the bronchial epithelium of mice [12] and gates the recruitment of neutrophils to the lung [13]. In healthy murine lung exposed to lipopolysaccharide (LPS), enhanced production of the neutrophil chemoattractant CXC-chemokine ligand 5 (CXCL5) and increased neutrophil recruitment are observed during the day. At night, endogenous glucocorticoids bind the glucocorticoid receptor (GR), inhibiting Cxcl5 transcription reducing neutrophil influx. Targeted ablation of Bmal1 in Club cells has a clear pro-inflammatory effect in this model [13]. Disruption of circadian rhythms in mice, to mimic chronic jet lag or shift work, cause an alteration in lung mechanics and clock gene expression in the lung in a sexually dimorphic manner [14]. Many genes expressed in the lung are under rhythmic circadian control and are involved in a vast number of processes [15].

## Immune Clock

Both the innate and adaptive immune systems oscillate in a circadian manner. Trafficking of immune cells, susceptibility to bacterial infections and septic shock, pattern recognition receptor expression, phagocytosis, secretion of cytokines and chemokines are all under rhythmic control [16–24].

Haspel et al. used a genome-wide approach to show that during acute endotoxemic lung injury in mice, there was an increase in rhythmic processes brought about through an up-regulation in newly rhythmic pathways. This suggests that a complex re-organization of cellular and molecular circadian rhythms occurs in acute lung injury and demonstrates the importance of circadian rhythm in disease processes [25].

## Clinical Translation

Recently, the importance of time of day in clinical practice has been realized [6, 26–35] (Table 1). The time of day at which our immune system is triggered, by for example sustaining a wound, undergoing surgery or having a vaccination, has a significant impact on how we respond.

**Table 1:** Summary of clinical studies demonstrating the importance of circadian clock biology

Clinical	Comments	References
Shift workers and regular jet-lag	Misalignment of internal clocks with environmental light–dark levels. Epidemiological studies show increased risks of: Cardiovascular disease, prostate cancer, lymphoma, breast cancer and colorectal cancer	[26–31]
Critically ill patients on ICU	Critically ill patients on ICU are nursed around the clock with no differentiation between night and day. Such patients with sepsis have impaired circadian melatonin rhythms	[32]
Chemotherapy infusions	Timing chemotherapy infusions with circadian rhythms in patients with metastatic colorectal cancer increased the effectiveness of chemotherapy and significantly reduced toxic side-effects compared to conventional constant-rate infusion	[33]
Vaccination	276 patients (over 65 years of age) vaccinated in the morning had greater antibody titers 1 month later than patients vaccinated in the afternoon.	[6]
Surgery	In patients undergoing aortic valve replacement, the incidence of major adverse cardiac events was lower in the afternoon surgery group than in the morning group. Perioperative myocardial injury was significantly lower in the afternoon group than in the morning group. Rev-Erbα antagonism may be a pharmacological strategy for cardioprotection	[34]
Wound healing	Skin wounds in mice wounded during the circadian rest period healed less quickly than those wounded during the active period. Analysis of a database of human burn injuries showed that those incurred during the night (rest period) healed more slowly than wounds acquired during the day (active period)	[35]

## Asthma

Asthma is a heterogenous disease usually characterized by chronic airway inflammation. It is defined by the history of respiratory symptoms such as wheeze, shortness of breath, chest tightness, and cough that vary over time and in intensity together with variable expiratory airflow limitation [36]. Asthma is a common disease affecting between 1 and 18% of the world’s population in different countries [36].

## Asthma and Circadian Rhythm

### Symptoms

Asthma is a disease with a strong circadian rhythm; it is characteristic of asthma that symptoms worsen in the early hours of the morning around 4:00 am [37]. Nocturnal symptoms in asthma are common and are an important indicator for escalation of treatment. Sudden death in asthma also tends to occur overnight [38].

### Airway Physiology

Physiological parameters of airway resistance, forced expiratory volume in 1 s (FEV_1_), and peak expiratory flow (PEF) are commonly measured in respiratory clinics and as outcome measures in drug trials. Both FEV_1_ and PEF vary in a circadian manner in healthy individuals with a nadir at approximately 4:00 am. However, in asthma, the amplitude of the circadian rhythm of both FEV_1_ and PEF is greatly magnified [39].

### Airway Inflammation

**Fig. 3:**
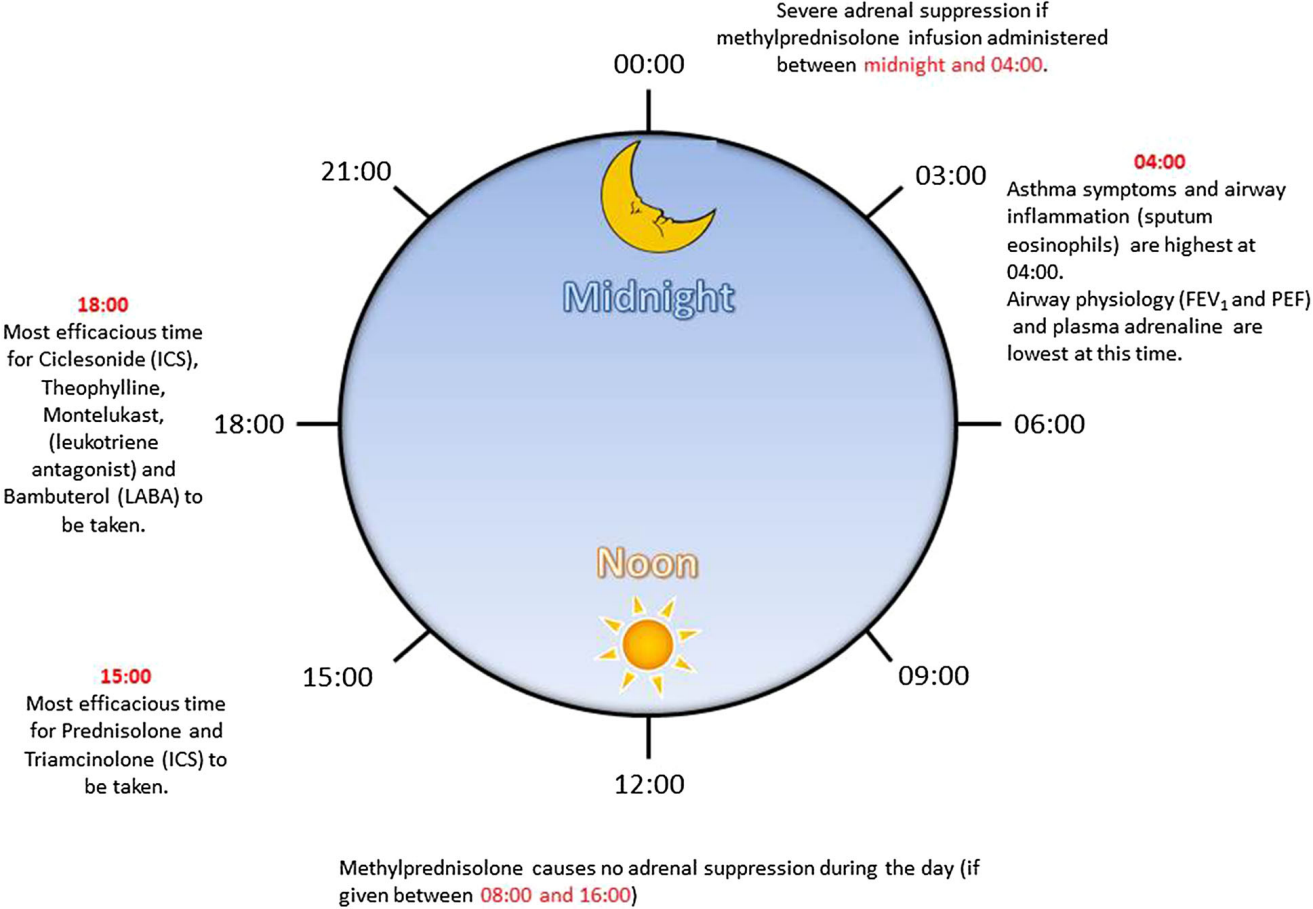
Asthma varies over 24 h. A summary of the changes that occur in asthma over 24 h. Airways become more narrowed at 4 am in asthma, coincident with increased symptoms and increased airway inflammation also at this time. The most efficacious time to take steroids (systemic and inhaled) may well be about 3 pm. There is no adrenal suppression of steroids are given between 8 am and 4 pm, but there is adrenal suppression if given between midnight and 4 am. Theophylline, leukotriene antagonists and LABAs may be more efficacious if taken in the early evening rather than the morning

There is a circadian variation in the number of alveolar eosinophils (significantly more present at 4:00 am versus 4:00 pm) in subjects with nocturnal asthma compared to those with nonnocturnal asthma, undergoing bronchoscopy [40] (Fig. 3). Nocturnal asthma probably represents a group of patients with poorly controlled asthma. However, results are somewhat conflicting [41] and further research in this area is needed.

### Molecular Clock

Ehlers et al. demonstrated a potential role for the clock gene *bmal1* in modulating viral exacerbations in asthma; *bmal1*^−*/*−^ mice developed extensive asthma-like airway changes post-viral infection, including mucus production and increased airway resistance [42]. Asthma is a disease with a strong time-of-day variability and therefore a chronotherapeutic approach to treatment might be highly beneficial for patients.

## Chronotherapy in Asthma

Chronotherapy is the synchronizing of drug concentrations to rhythms in disease activity, increasing efficacy as well as reducing adverse effects. The effectiveness of chronotherapy for asthma is most often determined by its effects on the morning dip in PEF and FEV_1_; other outcome measures are inflammation (bronchoalveolar or blood inflammatory cells, methacholine challenge PC_20_) and clinical outcome measures, such as quality of life, nocturnal wakenings, and exacerbations.

Current treatment guidelines do not reflect chronotherapy, phenotype, or endotype; rather they provide a linear treatment algorithm, based on asthma symptoms [43]. Inhaled corticosteroids (ICSs), with or without long-acting beta agonists (LABAs), are the mainstays of pharmacological treatment for mild-to-moderate asthma. Severe asthma is defined as asthma that requires treatment with high-dose ICSs plus a second controller and/or systemic corticosteroids to prevent it from becoming “uncontrolled” or that remains “uncontrolled” despite this therapy [44].

### Systemic Corticosteroids

There is a well-recognized endogenous circadian variation in cortisol levels, with levels of cortisol highest in the morning and lowest during the night. Infusion of methylprednisolone between 8:00 am and 4:00 pm caused no adrenal suppression; yet an infusion administered between 12:00 and 4:00 am caused severe adrenocortical suppression. Infusion during 4:00 and 8:00 pm and between 4:00 am and 8:00 am resulted in moderate adrenocortical suppression [45].

In a study investigating the best time of day to administer 50 mg prednisolone, three time points were looked at 8:00 am, 3:00 pm, or 8:00 pm and the outcome measure used was the decline in over-night FEV_1_ as well as broncholavage for inflammatory cells. Interestingly, 50 mg of prednisolone reduced the nocturnal decline in FEV_1_ only at 3:00 pm (in association with reduced neutrophils, eosinophils, lymphocytes and macrophages in the BAL), but was ineffective at 8:00 am or 8:00 pm [46]. These results are consistent with other studies, suggesting that synthetic corticosteroids administered at 3:00 pm are more effective in nocturnal asthma and cause less disruption to endogenous circadian cortisol rhythm [47].

### Inhaled Corticosteroids (ICSs)

ICSs are the most important treatment for asthma, controlling airway inflammation [48, 49]. Inhaling corticosteroids rather than taking them systemically reduces side effects. Several studies have investigated the chronotherapy of ICSs. Triamcinolone acetate taken at 3:00 pm (800 μg) was at least equivalent to a four-times-a-day (200 μg) treatment schedule. Blood eosinophils and methacholine challenge (PC_20_) were also measured but did not vary significantly between groups [50]. Pincus et al. showed that triamcinolone acetate taken either four times a day (800 μg/day) or as a single dose at 5:30 pm improved morning and evening PEF in a comparable manner, but not if taken as a single dose at 8:00 am. There was an improvement in methacholine challenge in all groups, but no difference between groups and this was also the case for nocturnal wakenings and quality of life indices, however there was a significant decrease in blood eosinophils in the four-times-a-day group compared to the other two groups [51]. A double-blind, randomized, parallel-group study of 209 asthmatic patients investigated the efficacy of taking 200 µg inhaled ciclesonide (Alvesco^®^, Teijin Pharma Ltd, Tokyo, Japan) in the morning or in the evening, for 8 weeks. Taking ciclesonide once daily in the evening improved the morning PEF better than if ciclesonide was taken only in the morning. However, morning and evening administration was equally effective for symptoms, use of rescue medication, and number of asthma exacerbations [52, 53]. These results are consistent with chronotherapeutic studies investigating oral corticosteroids.

### β2-Adrenergic Agonist Medication

β2-agonists (BAs) cause relaxation of airway smooth muscle, increasing airway diameter, and relieving bronchoconstriction; they are also anti-inflammatory [54]. Plasma adrenaline levels fluctuate in a circadian manner with a nadir at 4 am and a peak at 4:00 pm in both healthy and asthma subjects [55]. BAs are short-acting BAs (SABA) with duration of around 4 h, or long-acting (LABA) effective for 12–24 h. Procaterol (USAN) and fenoterol (Berotec—WBP) are SABAs that strongly induce the clock gene, hPer1, in human bronchial epithelial cells in vitro [56]. The chronotherapy of inhaled LABAs has not been extensively investigated and it would be interesting to investigate nighttime versus morning once-daily dosing with these agents.

The LABA tablet formulation Terbutaline (Bricanyl Depot^®^, AB Draco, Lund, Sweden) was administered to asthmatics in synchrony with the circadian rhythm of lung function; 5 mg given in the morning (8:00 am) and in the evening (8:00 pm) when lung function was beginning its decline. This chronotherapeutic strategy significantly increased the 24-h mean PEF and FEV_1_ and almost prevented the nocturnal decline [57–59]. Bambuterol (Bambec^®^, Astra Draco, Lund, Sweden), a prodrug of terbutaline, exerts a bronchodilator effect for 24 h. Evening dosing (20 mg) resulted in a considerably higher morning FEV_1_ and PEF compared to morning dosing.

Vilanterol (GlaxoSmithKline plc, United Kingdom), an ultra-LABA, acts for 24 h. There is no difference between morning or evening dosing with fluticasone furoate/vilanterol 100/25 μg, in patients with persistent asthma [60]; suggesting that the timing of dosing with ultra-LABAs is not important; however, any circadian effects of these long-acting drugs may well be masked.

### Anticholinergic Agents

Increased cholinergic tone through the vagus nerve at night may cause bronchoconstriction and mucus secretion [61]. The vagus may be one of the most important pathways for conveying circadian signals from the central clock to peripheral clocks in the respiratory tract [62]. Inhaled muscarinic antagonists are classified according to their duration of action; short-acting muscarinic antagonists (SAMAs) include ipratropium bromide and long-acting muscarinic antagonists (LAMAs) include tiotropium, aclidinium, and glycopyrronium. Inhaled anticholinergic agents produce inconsistent results in patients with nocturnal asthma [63, 64]. This may be because inadequate doses were used [61]. Several studies have shown that if large enough doses of anticholinergic medication are taken late at night or very early in the morning, the nocturnal decline in PEF in nocturnal asthmatics can be prevented [63–65]. Of the LAMAs, Tiotropium (Boehringer Ingelheim, Ingelheim am Rhein, Germany) showed no significant differences in effect on airway caliber when administered once daily in the morning versus evening [66]. However, the long duration of action of this high-affinity medication may mask possible circadian time-dependent effects.

## Leukotriene Receptor Antagonists

Montelukast (Merck), a leukotriene receptor antagonist, is recommended to be taken once daily in the evening. A double-blind study showed that montelukast better improved FEV_1_ when dosed in the evening than in the morning [67].

## Theophyllines

Theophyllines are anti-inflammatory agents. Once-daily preparations are dosed at night as this was shown to be more effective for increasing serum theophylline concentration at the time when lung function was worse, and this regimen improved both symptoms and PEF [68].

## Chronic Obstructive Pulmonary Disease (COPD)

COPD affects about 10% of people over 40 years of age, is a leading cause of hospital admissions, and is now the third-ranked cause of death worldwide [69]. The major risk factor for COPD in Western countries is cigarette smoking. COPD is characterized by progressive airflow obstruction, predominantly affecting the peripheral airways; this leads to air trapping, dynamic hyperinflation, and shortness of breath on exertion.

## COPD and Circadian Rhythm

In comparison to asthma, the link between circadian rhythm and COPD is less well established. Furthermore, there is a well-recognized overlap condition asthma-COPD overlap syndrome (ACOS), occurring in over 20% of COPD patients, in which features of both conditions exist concurrently [70, 71]. It is possible that ACOS influences the rhythmic findings in COPD studies.

### Symptoms

As in asthma, symptoms of COPD worsen in the morning [72]. In patients who experience morning symptoms, the most common morning symptoms were coughing, shortness of breath, and sputum production. Research has also indicated that patients who experience morning symptoms are at higher risk for exacerbations and are more likely to use their rescue inhaler [73]. The diurnal variation in symptom severity has been observed during COPD exacerbations, with elevated risk for intubation during early morning hours in the emergency department [74]. Importantly, a recent study showed that COPD patients that report both or either nocturnal or early morning symptoms have poorer health compared with patients who do not have a worsening of symptoms at specific times of day [75].

### Airway Physiology

There is a circadian variation in FEV_1_ in stable COPD that demonstrates a similar pattern, peaking at 4:00 pm and dipping at around 4:00 am, however the amplitude is substantially less than in asthmatic subjects [66, 76].

### Molecular Clock

Sirtuin1 (SIRT1), an NAD+-dependent deacetylase, affects clock function by binding with CLOCK:BMAL1 complexes and deacetylating BMAL1 and PER2 proteins [77, 78]. Rhythmic levels and activity of SIRT1 are reduced in mouse lungs exposed to cigarette smoke and in patients with COPD [79, 80]. This leads to BMAL1 acetylation and its enhanced degradation in mouse lungs [81].

A recent study by Sundar et al. shows a significant reduction of REV-ERBα in small airway epithelial cells taken from patients with COPD, increased inflammatory responses in REV-ERBα knockout (KO) mice as compared to wild-type mice post cigarette smoke exposure, and a significant increase in pro-inflammatory cytokines in the KO. These findings highlight REV-ERBα as an exciting target for clock-based treatment for COPD [82].

The use of E-cigarettes is becoming increasingly popular. Mice exposed to vapor from E-cigarettes demonstrate altered clock gene expression both systemically and in their lungs, as well as disruption of downstream signaling pathways [83].

Chronic CS exposure in mice combined with Influenza A Virus (IAV) infection altered the timing of clock gene expression and reduced locomotor activity in parallel with increased lung inflammation, disrupted rhythms of pulmonary function, and emphysema. BMAL1 KO mice infected with IAV showed pronounced detriments in behavior and survival, and increased lung inflammatory and pro-fibrotic responses. This suggests that remodeling of lung clock function following IAV infection alters clock-dependent gene expression and normal rhythms of lung function, enhanced emphysematous, and injurious responses [84].

## Chronotherapy in COPD

Guidelines for the treatment of COPD have consistently recommended long-acting inhaled bronchodilators—either LAMAs or LABAs—as initial maintenance therapy. If disease control is not achieved, as manifested by inadequate lung function and disease exacerbations, guidelines recommend their combined use [85]. Although there is general agreement about the role of LAMAs and LABAs in the treatment of COPD, the role for inhaled glucocorticoids in this treatment guideline has been the object of much debate because of their modest effectiveness and concerns about safety, particularly the risk of pneumonia [86]. The recent Global Initiative for Chronic Obstructive Lung Disease (GOLD) guidelines recommend that the addition of an inhaled glucocorticoid be limited to patients with severe loss of lung function and those with frequent exacerbations [85].

## LABA and LAMA

Currently, two types of inhaled long-acting bronchodilators are commonly utilized in COPD: LABA (formoterol, salmeterol) with duration of action of 12 h, and a LAMA (tiotropium) with duration of action > 24 h [66, 87].

In a study looking at tiotropium as a single agent either in the morning or evening, there was an overall increase in FEV_1_ throughout the day in both groups with no significant difference in the improvement in night-time dip between the two groups [66]. van Noord et al. reported that a maintenance therapy of combined tiotropium and formoterol, both once-daily, provided additive effects on FEV_1_ throughout the 24 h in patients with COPD [88]. Add-on therapy of formoterol in the morning to maintenance therapy of tiotropium significantly improved FEV_1_, forced vital capacity (FVC) and inspiratory capacity (IC) in COPD. A second formoterol dose in the evening provided a further improvement in average FEV_1_, FVC, and IC during the night-time hours [89]. In a randomized, blind, crossover study, five different treatments were compared: tiotropium in the morning, tiotropium in the morning and formoterol at night, formoterol twice daily, tiotropium in the morning and formoterol twice daily, and lastly, formoterol twice daily and tiotropium at night. In patients with moderate to severe COPD, combination therapy with tiotropium administered in the morning (and formoterol twice daily) was the most effective; in patients with prevailing night-time symptoms, treatment with tiotropium in the evening (and formoterol twice daily) reduced symptoms and use of salbutamol and showed less variability of FEV_1_ during the 24 h [90]. This suggests that the timing of tiotropium should be guided by the presence of night-time symptoms.

More recently, once-daily dosing with tiotropium-like drugs such as umeclidinium (GlaxoSmithKline) and glycopyrronium (Novartis Pharmaceuticals UK Ltd) are being used in clinical practice. To date, there are no chronotherapy studies using these agents.

### Theophyllines

Three regimens of sustained-release theophylline (SRT), Theostat, were administered in a randomized cross-over trial. In the first, a high dose (8 mg/kg) in the morning, and a low dose (4 mg/kg) at night, in the second intermediate dosing (6 mg/kg) morning and evening, and in the third low dose in the morning and high dose in the evening. Serial FEV_1_ measurements demonstrated that unequal, twice-daily SRT dosing with the greater amount of drug at night was the most beneficial in the treatment of COPD [91].

## Discussion

Asthma varies considerably throughout the day, and to a lesser extent so does COPD. The rhythmic variation in symptoms, airway physiology, and inflammation point to a role for the molecular clock in the pathogenesis of both these inflammatory airway diseases. Although there have been many small chronotherapeutic trials, particularly in asthma, the findings are not always being put into practice, particularly when it comes to prescribing systemic and inhaled corticosteroids, which may be more effective if taken in the afternoon rather than in the morning. Future large, randomized clinical trials are needed to inform clinical practice.

Poor adherence to inhaled corticosteroid is a major obstacle in the management of asthma [92]; how chronotherapy would impact adherence rates is an important area for future research. Delayed release preparations might allow the drug to be taken in the morning, but only become active later in the day.

Our growing understanding of how components of the molecular clock interact with critical elements of inflammatory pathways offers the exciting potential for the use of pharmacological agents that target these clock proteins as anti-inflammatory agents. The challenge now is to produce high-affinity, high-efficacy molecules that enhance the activity of clock proteins. Topically delivered, clock-acting compounds might allow selective manipulation of the pulmonary clock.

Elucidating the molecular pathways that precede the diurnal worsening of symptoms may provide a major advance in treatment options for patients with asthma. Targeting a well-defined circadian molecular pathway at a predictable time point, when the pathway is upregulated, will result in more efficacious therapies with fewer side effects.

## Electronic supplementary material

Supplementary material, approximately 228 KB.
