# Supplementary material for: The Role of the Body Clock in Asthma and COPD: Implication for Treatment
Source: Pulm Ther. 2018 Jun 1;4(1):29–43. doi: 10.1007/s41030-018-0058-6 (PMC6967276; doi:10.1007/s41030-018-0058-6)
Supplement: Supplementary file 1 — Supplementary material, approximately 228 KB. [file 41030_2018_58_MOESM1_ESM.pdf]

Pulmonary Therapy

OPEN ACCESS

PEER-REVIEWED INFOGRAPHIC

The Role of the Body Clock in Asthma and COPD: Implication for Treatment

Cite this article as:Krakowiak, K. & Durrington, H.J. Pulm Ther (2018) 4: 29. https://doi.org/10.1007/s41030-018-0058-6

INDICATION

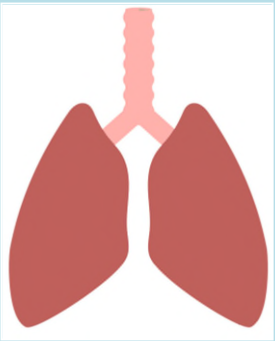

Asthma and COPD

BACKGROUND

Over the past 15 years, our understanding of the body clock and **biological rhythms** has increased immeasurably. Several **inflammatory diseases**, such as asthma, display a marked time of day pattern in symptoms.

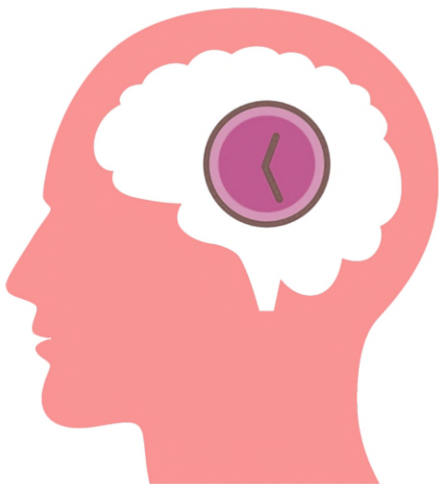

THERAPY

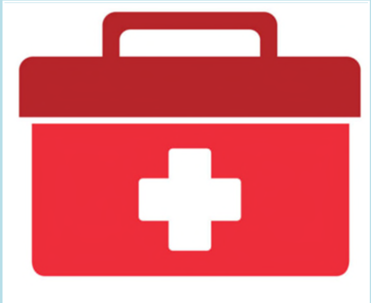

Various

AIM

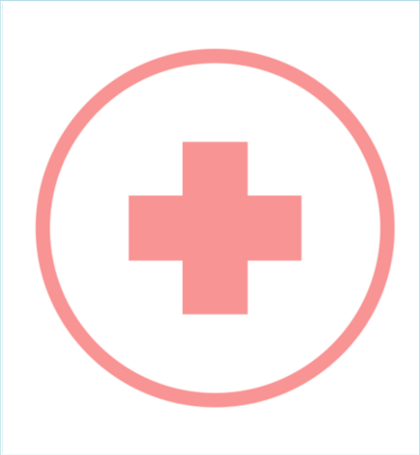

This review aimed to discuss recent advances in the understanding of **circadian biology** and how this relates to the treatment and management of **asthma** and **COPD**.

Article Summary

'Chronotherapy' is the timing of drug administration to the biological rhythm of disease and is important in both **asthma** and **COPD**.

Asthma displays strong **circadian variation** in symptoms, airway physiology and airway inflammation.

To a lesser extent, **COPD** also shows **rhythmic variation** in symptoms and airway physiology.

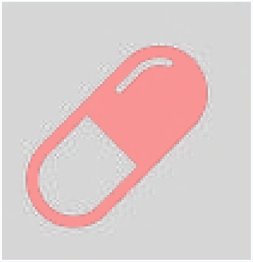

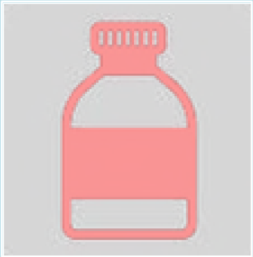

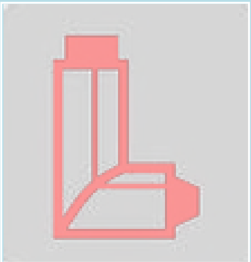

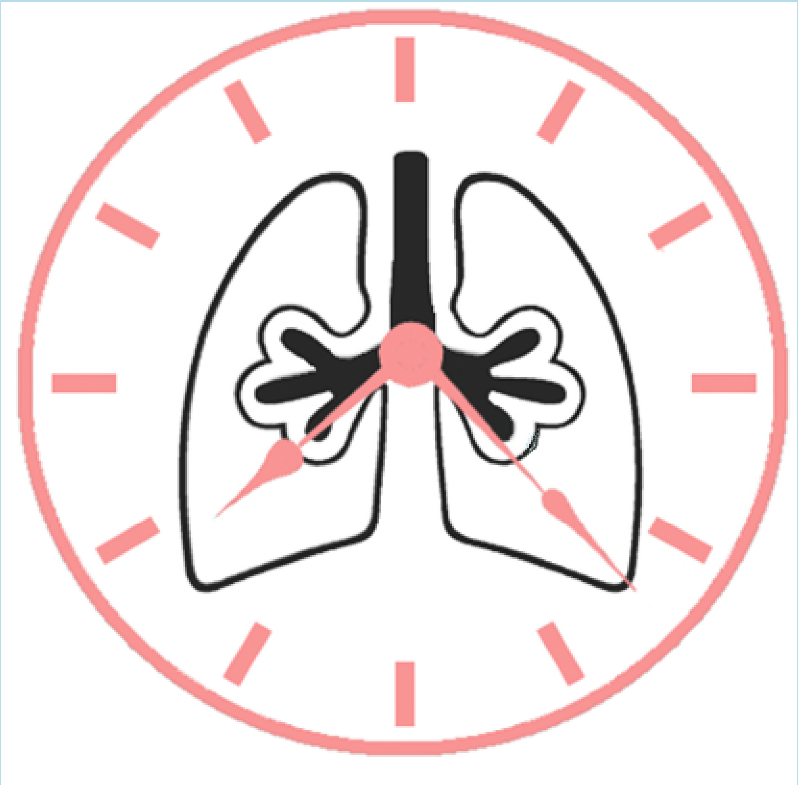

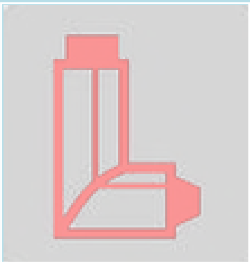

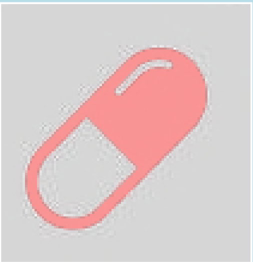

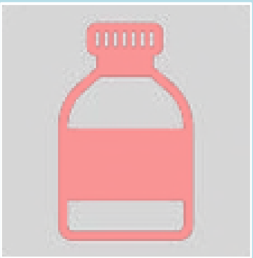

Future, large, randomised controlled **clinical trials** are needed to confirm the results of smaller studies that the best time of day to take **systemic** or **inhaled corticosteroids** is actually in the afternoon and not the morning.

The circadian molecular clock is important in the **pathogenesis** of both Asthma and COPD.

Abbreviations:

COPD = Chronic Obstructive Pulmonary Disease

Adis

This infographic represents the opinions of the authors. For a full list of acknowledgments and disclosures for all authors of this article, please see the full text online.

© The Authors, CC-BY-NC 2020
